# How to review a case report

**DOI:** 10.1186/s13256-016-0853-3

**Published:** 2016-04-06

**Authors:** Rakesh Garg, Shaheen E. Lakhan, Ananda K. Dhanasekaran

**Affiliations:** 1 Department of Anaesthesiology, Pain and Palliative Care, DR BRAIRCH, AIIMS, Ansari Nagar, New Delhi, 110029 India; 2 Neurology and Medical Education, California University of Science and Medicine - School of Medicine, Colton, CA USA; 3 Sandwell & West Birmingham Hospitals, NHS Trust, Birmingham, UK

## Introduction

Sharing individual patient experiences with clinical colleagues is an essential component of learning from each other. This sharing of information may be made global by reporting in a scientific journal. In medicine, patient management decisions are generally based on the evidence available for use of a particular investigation or technology [1]. The hierarchical rank of the evidence signifies the probability of bias. The higher up the hierarchy, the better its reliability and thus its clinical acceptance (Table 1). Though case reports remain lowest in the hierarchy of evidence, with meta-analysis representing the highest level, they nevertheless constitute important information with regard to rare events and may be considered as anecdotal evidence [2] (Table 1). Case reports may stimulate the generation of new hypotheses, and thus may support the emergence of new research.

**Table 1:** Levels of evidence

Level of evidence	Type of evidence
1a	Evidence obtained from meta-analysis of randomized trials
1b	Evidence obtained from at least one randomized trial
2a	Evidence obtained from one well-designed controlled study without randomization
2b	Evidence obtained from at least one other type of well-designed quasi-experimental study
3	Evidence obtained from well-designed non-experimental studies, such as comparative studies, correlation studies, and case reports
4	Evidence obtained from expert committee reports or opinions or clinical experience of respected authorities

Produced by Bob Phillips, Chris Ball, Dave Sackett, Doug Badenoch, Sharon Straus, Brian Haynes, Martin Dawes since November 1998. Updated by Jeremy Howick March 2009. Assessed from: http://www.cebm.net/oxford-centre-evidence-based-medicine-levels-evidence-march-2009. Assessed on 1 Nov 2015

The definition of a case report or a case series is not well defined in the literature and has been defined variously by different journals and authors. However, the basic definition of a case report is the detailed report of an individual including aspects like exposure, symptoms, signs, intervention, and outcome. It has been suggested that a report with more than four cases be called a case series and those with fewer than four a case report [3]. A case series is descriptive in design. Other authors describe “a collection of patients” as a case series and “a few patients” as a case report [4]. We suggest that should more than one case be reported, it may be defined as a case series—a concept proposed by other authors [5].

## The importance of case reports

A case report may describe an unusual etiology, an unusual or unknown disorder, a challenging differential diagnosis, an unusual setting for care, information that can not be reproduced due to ethical reasons, unusual or puzzling clinical features, improved or unique technical procedures, unusual interactions, rare or novel adverse reactions to care, or new insight into the pathogenesis of disease [6, 7]. In recent years, the publication of case reports has been given low priority by many high impact factor journals. However, the need for reporting such events remains. There are some journals dedicated purely to case reports, such as the *Journal of Medical Case Reports*, emphasizing their importance in modern literature. In the past, isolated case reports have led to significant advancements in patient care. For example, case reports concerning pulmonary hypertension and anorexic agents led to further trials and the identification of the mechanism and risk factors associated with these agents [2, 8].

## Reporting and publishing requirements

The reporting of cases varies for different journals. The authors need to follow the instructions for the intended publication. Owing to significant variability, it would be difficult to have uniform publication guidelines for case reports. A checklist called the CARE guidelines is useful for authors writing case reports [9, 10]. However, it would be universally prudent to include a title, keywords, abstract, introduction, patient information, clinical findings, timeline, diagnostic assessment, therapeutic interventions, follow-up and outcomes, discussion, patient perspective, and informed consent.

## Peer review process

The peer review process is an essential part of ethical and scientific writing. Peer review ultimately helps improve articles by providing valuable feedback to the author and helps editors make a decision regarding publication. The peer reviewer should provide unbiased, constructive feedback regarding the manuscript. They may also highlight the strengths and weaknesses of the report. When reviewing an article, it is prudent to read the entire manuscript first to understand the overall content and message. The reviewer than may read section-wise and provide comments to the authors and editorial team accordingly. The reviewer needs to consider the following important points when reviewing a case for possible publication [8, 9] (summarized in Table 2).

**Table 2:** Checklist for case report reviewer

Section	Topic	Met or Unmet
General	Novelty	
	Patient consent	
	Ethical practice as per standard of care	
Title	Truly describes the core message of the case. Includes the phrase “a case report.”	
Abstract	Incorporates the core key message with necessary detail in a concise manner.	
Key words	Medical Subject Headings (MeSH) keywords, core message included	
Introduction	Emphasizes need of publication by novelty of the case or the specific adverse event.	
Case description	Appropriate details of the case, including demography, assessment, findings, investigations and so on. Mentions intervention in detail or describes the dose, timing, and route of drugs.	
Discussion	Emphasizes why the case is important to medicine. Adequate literature review pertinent to the case. Mentions the limitations related to the case.	
Conclusion	Implication of case with a core key message.	
Recommendation	Reject/minor revision/major revision/accept as submitted	

### Novelty

Novelty remains the foremost important aspect of a case. The case report should introduce novel aspects of patient evaluation, investigation, treatment, or any other aspect related to patient care. The relevant information becomes a hypothesis generator for further study. The novelty may at times be balanced with some important information like severe adverse effects, even if they have been reported earlier. Reporting adverse events remains important so that information on cumulative adverse effects can be gathered globally, which helps in preparing a policy or guideline or a warning note for its use in patients. The data related to adverse effects include not only the impact but also the number of patients affected. This becomes more important for serious adverse effects. In the absence of an international registry for adverse effects, published case reports are important pieces of information. Owing to ethical concerns, formal evaluation may not be feasible in the format of prospective study.

### Essential description

The case needs to have all essential details to allow a useful conclusion to emerge. For example, if a case is being reported for hemodynamic variability due to a drug, then the drug dose and timing along with timed vital signs need to be described.

### Authenticity and genuineness

Honesty remains the most important basic principle of all publications. This remains a primary responsibility of the authors. However, if there is any doubt, reviewers may seek clarification. This doubt may result from some discordance in the case description. At times, a lack of correlation between the figures and description may act as “red flags.” For instance, authors may discuss a technique for dealing with a difficult airway, but the figure is of a normal-appearing airway. Another example would be where the data and figure do not correlate in a hemodynamic response related to a drug or a technique, with the graphical picture or screenshot of hemodynamics acting as an alert sign. Such cause for concern may be communicated in confidence to the editor.

### Ethical or competing interests

Ethical issues need to be cautiously interpreted and communicated. The unethical use of a drug or device is not desirable and often unworthy of publication. This may relate to the route or dose of the drug administered. The off-label use of drugs where known side effects are greater than potential benefit needs to be discouraged and remains an example of unethical use. This use may be related to the drug dose, particularly when the drug dose exceeds the routine recommended dose, or to the route of administration. As an example, the maximal dose of acetaminophen (paracetamol) is 4g/day, and if an author reports exceeding this dose, it should be noted why a greater than recommended dose was used. Ultimately, the use of a drug or its route of administration needs to be justified in the manuscript. The reviewers need to serve as content experts regarding the drugs and other technologies used in the case. A literature search by the reviewer provides the data to comment on this aspect.

Competing interests (or conflicts of interest) are concerns that interfere or potentially interfere with presentation, review, or publication. They must be declared by the authors. Conflicts can relate to patient-related professional attributes (like the use of a particular procedure, drug, or instrument) being affected by some secondary gains (financial, non-financial, professional, personal). Financial conflict may be related to ownership, paid consultancy, patents, grants, honoraria, and gifts. Non-financial conflicts may be related to memberships, relationships, appearance as an expert witness, or personal convictions. At times, the conflict may be related to the author’s relationship with an organization or another person. A conflict may influence the interpretation of the outcome in an inappropriate and unscientific manner. Although conflicts may not be totally abolished, they must be disclosed when they reasonably exist. This disclosure should include information such as funding sources, present membership, and patents pending. Reviewers should cautiously interpret any potential bias regarding the outcome of the case based on the reported conflicts. This is essential for transparent reporting of research. At times, competing interests may be discovered by a reviewer and should be included in comments to the editorial team. Such conflicts may again be ascertained when the reviewer reviews the literature during the peer review process. The reviewer should also disclose their own conflicts related to the manuscript review when sending their report to the editorial team.

### Impact on clinical practice

This is an important aspect for the final decision of whether to publish a case report. The main thrust or carry-home message needs to be emphasized clearly. It needs to be elaborated upon in concluding remarks.

### Patient anonymity, consent, and ethical approval

When reviewing the manuscript of a case report, reviewers should ensure that the patient’s anonymity and confidentiality is protected. The reviewers should check that patient identifiers have been removed or masked from all aspects of the manuscript, whether in writing or within photograph. Identifiers can include things like the name of the patient, geographical location, date of birth, phone numbers, email of the patient, medical record numbers, or biometric identifiers. Utmost care needs to be taken to provide full anonymity for the patient.

Consent is required to participate in research, receive a certain treatment, and publish identifiable details. These consents are for different purposes and need to be explained separately to the patient. A patient’s consent to participate in the research or for use of the drug may not extend to consent for publication. All these aspects of consent must be explained to the patient, written explicitly in the patient’s own language, understood by the patient, and signed by the patient. For the purpose of the case, the patient must understand and consent for any new technique or drug (its dose, route, and timing) being used. In the case of a drug being used for a non-standard indication or route, consent for use must also be described. Patient consent is essential for the publication of a case if patient body parts are displayed in the article. This also includes any identifiers that can reveal the identity of the patient, such as the patient’s hospital identification number, address, and any other unique identifier. In situations where revealing the patient’s identity cannot be fully avoided, for example if the report requires an image of an identifiable body part like the face, then this should be explained to the patient, the image shown to them, and consent taken. Should the patient die, then consent must be obtained from next of kin or legal representative.

With case series, securing individual patient consent is advised and preferable. The authors may also need institutional review board (IRB) approval to publish a case series. IRBs can waive the need for consent if a study is conducted retrospectively and data are collected from patient notes for the purpose of research, usually in an anonymized way. However, wherever possible, individual patient consent is preferable, even for a retrospective study. Consent is mandatory for any prospective data collection for the purpose of publication as a case series. Consent and/or IRB approval must be disclosed in the case report and reasons for not obtaining individual consent may be described, if applicable.

There may be situations in which publishing patient details without their consent is justified, but this is a decision that should be made by the journal editor, who may decide to discuss the case with the Committee on Publication Ethics. Reviewers need to emphasize the issue to the editor when submitting their comments.

### Manuscript writing

The CARE guidelines provide a framework that supports transparency and accuracy in the publication of case reports and the reporting of information from patient encounters. The acronym CARE was created from CA (the first two letters in “case”) and RE (the first two letters in “reports”). The initial CARE tools are the CARE checklist and the Case Report Writing Templates. These tools support the writing of case reports and provide data that inform clinical practice guidelines and provide early signals of effectiveness, harms, and cost [10].

The presentation of the case and its interpretation should be comprehensive and related. The various components of the manuscript should have sufficient information for understanding the key message of the case. The reviewer needs to comment on the relevant components of the manuscript. The reviewer should ascertain that the title of the case manuscript is relevant and includes keywords related to the case. The title should be short, descriptive, and interesting. The abstract should be brief, without any abbreviations, and include keywords. It is preferable to use Medical Subject Headings (MeSH) keywords. Reviewers must ensure that the introduction emphasizes the context of the case and describes the relevance and its importance in a concise and comprehensive manner. The case description should be complete and should follow basic rules of medical communication. The details regarding patient history, physical examination, investigations, differential diagnosis, management, and outcome should be described in chronological order. If repeated observations are present, then they may be tabulated. The use of graphs and figures helps the readers to better understand the case. Interpretation or inferences based on the outcomes should be avoided in this section and should be considered a part of the discussion. The discussion should highlight important aspects of the case, with its interpretation within the context of the available literature. References should be formatted as per the journal style. They should be complete and preferably of recent publications.

## Reviewer responsibility

The reviewer’s remarks are essential not only for the editorial team but also for authors. A good peer review requires honesty, sincerity, and punctuality. Even if a manuscript is rejected, the authors should receive learning points from peer review commentary. The best way to review a manuscript is to read the manuscript in full for a gross overview and develop general comments. Thereafter, the reviewer should address each section of the manuscript separately and precisely. This may be done after a literature search if the reviewer needs to substantiate his/her commentary.

### Constructive criticism

The reviewer’s remarks should be constructive to help the authors improve the manuscript for further consideration. If the manuscript is rejected, the authors should have a clear indication for the rejection. The remarks may be grouped as major and minor comments. Major comments likely suggest changes to the whole presentation, changing the primary aim of the case report, or adding images. Minor comments may include grammatical errors or getting references for a statement. The editorial team must be able to justify their decision on whether or not to accept an article for publication, often by citing peer review feedback. It is also good style to tabulate a list of the strengths and weaknesses of the manuscript.

### Fixed time for review

Reviewer remarks should be submitted within a specified timeframe. If any delay is expected, it should be communicated to the editorial team. Reviewers should not rush to submit feedback without sufficient time to adequately review the paper and perform any necessary literature searches. Should a reviewer be unable to submit the review within the specified timeframe, they should reply to the review invitation to decline at their earliest convenience. If, after accepting a review invitation, the reviewer realizes they do not have time to perform the review, this must be communicated to the editorial team.

### Conflict of interest

The reviewer’s conflicts of interest should be included along with the review. The conflicts may be related to the contents of the case, drugs, or devices pertaining to the case; the author(s); or the affiliated institution(s) of the author(s).

### Lack of expertise

The reviewer may decline to review the manuscript if they think the topic is out of their area of expertise. If, after accepting an invitation to review, the reviewer realizes they are unable to review the manuscript owing to a lack of expertise in that particular field, they should disclose the fact to the editorial team.

### Confidentiality

The reviewer should keep the manuscript confidential and should not use the contents of the unpublished manuscript in any form. Discussing the manuscript among colleagues or any scientific forum or meetings is inappropriate.

### Review of revised manuscript

At times, a manuscript is sent for re-review to the reviewer. The reviewer should read the revised manuscript, the author’s response to the previous round of peer review, and the editorial comments. Sometimes, the authors may disagree with the reviewer’s remarks. This issue needs to be elaborated on and communicated with the editor. The reviewer should support their views with appropriate literature references. If the authors justify their reason for disagreeing with the viewer, then their argument should be considered evidence-based. However, if the reviewer still requests the revision, this may be politely communicated to the author and editor with justification for the same. In response to reviewers remarks, authors may not agree fully and provide certain suggestion in the form of clarification related to reviewers remarks. The reviewers should take these clarifications judiciously and comment accordingly with the intent of improving the manuscript further.

## Conclusion

Peer reviewers have a significant role in the dissemination of scientific literature. They act as gatekeepers for science before it is released to society. Their sincerity and dedication is paramount to the success of any journal. The reviewers should follow a scientific and justifiable methodology for reviewing a case report for possible publication. Their comments should be constructive for the overall improvement of the manuscript and aid the editorial team in making a decision on publication. We hope this article will help reviewers to perform their important role in the best way possible. We send our best wishes to the reviewer community and, for those who are inspired to become reviewers after reading this article, our warm welcome to the reviewers’ club.
